# Retinal integrity in human babesiosis: a pilot study

**DOI:** 10.1186/s12886-024-03568-6

**Published:** 2024-07-24

**Authors:** Elyssa Dionne, Ron A. Adelman, Osman Cekic, Marjorie Golden, Anne Spichler Moffarah, Peter J. Krause, Shelli F. Farhadian

**Affiliations:** 1 Yale School of Medicine, 135 College Street, New Haven, CT 06510 USA; 2 Department of Ophthalmology and Visual Science, Yale University School of Medicine, 40 Temple Street, New Haven, CT 06510 USA; 3 Department of Ophthalmology, Mayo Clinic, Jacksonville, 4500 San Pablo Rd S, 32224 Jacksonville, Florida, FL USA; 4 Department of Internal Medicine: Infectious Disease, Yale School of Medicine, 333 Cedar Street, New Haven, CT 06510 USA; 5 Department of Epidemiology of Microbial Diseases, Yale School of Public Health, 60 College Street, New Haven, CT 06520 USA

**Keywords:** Babesiosis, Retinopathy, Pilot study

## Abstract

**Background:**

Prior case reports and animal studies have reported on potential ophthalmologic complications of babesiosis, but this issue has not previously been addressed in a cohort of patients with babesiosis. This cross-sectional descriptive pilot study evaluated the retinas of patients with acute babesiosis to determine if retinal abnormalities are a feature of the disease.

**Methods:**

We screened all patients admitted to Yale New Haven Hospital with laboratory confirmed babesiosis during the summer of 2023 and obtained informed consent. Patients were interviewed and underwent pupil dilation and a retinal examination using an indirect ophthalmoscope. Demographic and clinical information were obtained by questionnaire and through chart review.

**Results:**

Ten patients underwent retinal eye exams with results that were generally unremarkable. No study patients showed any signs of retinal inflammation, infection, retinal bleeding, retinal tears, or abnormal vessel formation that could be attributed to infection.

**Conclusion:**

This small study did not find evidence of retinopathy in patients with babesiosis. Further studies with larger populations, repeated exams, and long term follow up will further elucidate the potential small vessel complications of human babesiosis.

## Background

Babesiosis is a globally emerging tick-borne disease that is caused by *Babesia* spp., most commonly, *Babesia microti* [1]. Over the last decase, the number of babesiosis cases have more than doubled, and is predicted to continue to rise [2]. While it is estimated that 20% of adults and 40% of children who contract babesiosis are asymptomatic, some in the population, particularly those who are immunocompromised, can develop severe symptoms and complications, including cardiac, neurological, pulmonary, renal, and hepatic impairment and fatal infection [3].

Few studies have reported on potential ophthalmologic complications of babesiosis in humans. Case reports have described retinal nerve fiber layer infarcts, papillitis, splinter hemorrages, and one white centered hemorrhage [4, 5]. *Babesia canis* has been reported to cause ocular complications in dogs [6]. Studies of *Babesia bovis* infection in cattle demonstrate that cytoadherence of infected red blood cells, white blood cells, and platelets to vascular endothelium causes vascular obstruction in the brain, a similar mechanism to that described in the two human ocular cases [7]. However, it is unknown whether retinal abnormalities are a common feature of human babesiosis, and whether they are associated with the severity of babesiosis. We previously reported a high frequency of neurologic symptoms and renal impairment in hospitalized patients with babesiosis, suggesting small vessel occlusion as a possible biological mechanism underpinning these symptoms [8]. Since retinal abnormalities can reflect cerebral small vessel occlusion in other conditions, we aimed to assess for retinal abnormalities in hospitalized patients with babesiosis [9, 10]. We conducted a cross-sectional descriptive pilot study of patients hospitalized with acute babesiosis in a Connecticut hospital during the summer of 2023 to determine whether retinal abnormalities are a feature of babesiosis.

## Methods

We carried out a cross-sectional descriptive pilot study to assess presence or absence of retinopathy in babesiosis patients. The Institutional Review Board approved the study. All patients admitted to Yale New Haven Hospital with a confirmed diagnosis of babesiosis in the summer of 2023 were eligible for retinal screening. Inclusion criteria were a diagnosis of babesiosis based on blood smear and/or *B. microti* PCR; age over 18 years; and ability to provide informed consent. Once identified, patients were asked to sign an informed consent, were interviewed, and underwent pupil dilatation with phenylephrine and tropicamide. After a half hour to allow proper dilation, an expereinced retina specialist (initials OC) performed a bedside retinal exam using an indirect ophthalmoscope. Demographic and clinical information was obtained by questionnaire and through chart review. Peak parasitemia was determined through daily blood smears from the day of admission to discharge.

## Results

Twenty-seven babesiosis patients were admitted to Yale New Haven Hospital (YNHH) between June 25, 2023 and September 1, 2023. Twelve of them consented to participate in the study, and ten underwent retinal exams. The demographic and clinical information of the study participants is as listed in Table 1. The median age of study participants was 72 years (range 54–85). Four of ten patients were immunocompromised. Five of the ten study patients reported neurological symptoms, including five patients with episodes of impaired consciousness and three with concurrent headache. No patients reported visual impairment. All patients were treated with azithromycin and atovaquone. Six patients with Lyme disease coinfection or suspected coinfection were also treated with doxycycline, and three of them were later confirmed as having had Lyme disease coinfection. Two patients required blood transfusion and two had exchange transfusion (Table 1).

**Table 1:** Demographics, exams, and treatments of study patients

Study number	Age	Sex	Immunocompromised	Peak parasitemia (%)	Coinfection	Clinical Complications	Eye exam results	Ophthalmologic history	Babesiosis treatment	Ophthalmology consultation
**1**	74	F	no	7.2	none	renal impairment	Normal exam	dry eye, cataract extract and lens replacement in left eye	RBC transfusion, atovaquone + azithromycin	N
**2**	71	F	asplenic	25	none	none	tortuous vessels looks like high blood pressure	bilateral refractive surgery; cataract extract	atovaquone + azithromycin	N
**3**	82	F	no	< 1%	none	acute respiratory distress syndrome, liver injury, supraventricular tachycardia	Cataract 1 + nuclear sclerosis posterior virtuous detachment Altered pigmentation around periphery Subconjunctival hemorrhage PVD tilted disk pigment alterations around retina periphery	no known issues	atovaquone + azithromycin	N
**4**	85	M	Chronic inflammatory demyelinating polyneuropathy	7.2	lyme	supraventricular tachycardia	natural aging, tortuous vessels from HTN,	cataract surgery	atovaquone + azithromycin	N
**5**	65	M	no	< 1%	none	none	slight macular degeneration (consistent with family history), tortuous vessels in both eyes (consistent with HTN but no HTN)	slight macular degeneration	atovaquone + azithromycin	N
**6**	54	M	no	19	none	renal impairment	Normal exam	dry eye, cataract extract and lens replacement in left eye	RBC transfusion, atovaquone + azithromycin	N
**7**	56	M	asplenic	24.2	none	none	Normal exam	blurry vision	atovaquone + azithromycin	N
**8**	68	M	no	14	Lyme	none	Normal exam	no known issues	atovaquone + azithromycin	N
**9**	73	M	asplenic	30.4	Lyme	none	peripheral hyperpigmentation probably due to aging	Trabeculectomy	atovaquone + azithromycin	N
**10**	74	M	no	9.2	Lyme	septic shock	tortuous form HTN	cataracts, floaters, cataract surgery	atovaquone + azithromycin	N

RBC = red blood cells, PVD = peripheral vascular disease, HTN = hypertension

Retinal examination was generally unremarkable. No study patients showed any signs of retinal inflammation, infection, retinal bleeding, retinal tears, or abnormal vessel formation that could be attributed to infection. Three patients presented with tortuous vessels likely secondary to hypertension, which was previously diagnosed in two out of these patients. Two patients had altered peripheral pigmentation, which was thought to be due to the aging processes. Four patients also had a history of cataract extraction. One study patient presented with nuclear sclerosis, posterior vitreous detachment, altered pigmentation in the retinal periphery, subconjunctival hemorrhage of the left eye, and tilted disk. This patient had hypertension, hypercholesterolemia, hyperlipidemia, Lyme disease coinfection, and an abdominal aortic dissection, leading to her death 6 days after her eye examination.

## Discussion

This pilot study assessed the potential for retinopathy in patients with acute babesiosis who were admitted to YNHH, and is the largest cross-sectional ophthalmologic study of patients with babesiosis. The hospital is located in a region highly endemic for this tick-borne disease. None of the patients demonstrated evidence of retinopathy due to babesiosis.

We recently detected a high rate of neurological symptoms in hospitalized patients with babesiosis, and a strong association between neurological symptoms and both high parasite load and impaired renal function [8]. In that case series of babesiosis patients admitted to YNHH, 6% described transient vision loss, though few underwent a formal ophthalmologic examination. Our findings suggest that central nervous system small vessel occlusion is not a common feature of babesiosis and that alternative mechanisms may underlie neurological impairment that has been reported in over half of patients with acute babesiosis.

It has been hypothesized that *B. microti* parasites may cause microvascular obstruction through cytoadherence of infected red blood cells [11, 12]. This hypothesis is supported by the demonstration of cytoadherence and vascular obstruction in *B. bovis* and *B. canis*-infected red blood cells in cattle and dogs, respectively. The variable *B. bovis ves* gene family encodes for adhesion proteins and is analogous to *var* gene family in *Plasmodium falciparum*, a parasite that causes malaria [9]. Malaria is associated with sequestration of infected red blood cells in retinal and cerebral microvasculature, causing vessel obstruction. Indeed, prior cases of babesiosis-associated retinal nerve fiber layer infarcts further suggests the possibility of small vessel obstruction [4, 5]. We found no signs or symptoms of microvascular obstruction in the eyes of any patients, however, the number of study subjects was small. Moreover, our study was limited to a single indirect opthalmoscopic examination in the hospital. While this method of exam can be quite effective in assessing retinal abnormalities, it is possible that subtle changes would have been detected on fluorescein angiopgrapy and optical coherenece tomography angiography (OCT). The necessity of a bedside exam lead to a barrier of transport of the patients to the ophthalmology clinic and thus lack of accessible technology may have hindered our findings. We also would not have captured any long-term, or later ocular complications.

## Conclusion

This pilot study investigated the potential for retinopathy in patients with *B. microti* babesiosis during one summer season in Connecticut. Ten patients underwent dilated eye examination at the bedside, and none of these patients showed indications of retinopathy that could be attributed to babesiosis. Our sample size was too small to conclusively exclude retinopathy as a pathogenic feature of babesiosis. Future studies should include a larger patient population, more in depth retial exams, repeated examinations, and long term follow-up of study patients.

## Data Availability

The datasets generated and/or analysed during the current study are not publicly available due private health information but are available from the corresponding author on reasonable request.

## Abbreviations

YNHH: Yale New Haven Hospital
